# Eosinophil Activation Status in Separate Compartments and Association with Asthma

**DOI:** 10.3389/fmed.2017.00075

**Published:** 2017-06-12

**Authors:** Mats W. Johansson

**Affiliations:** ^1^Department of Biomolecular Chemistry, University of Wisconsin, Madison, WI, United States

**Keywords:** eosinophils, activation, asthma, integrins, interleukin-5

## Abstract

Asthma is frequently characterized by eosinophil-rich airway inflammation. Airway eosinophilia is associated with asthma exacerbations and likely plays a part in airway remodeling. Eosinophil recruitment from the bloodstream depends on circulating eosinophils becoming activated, which leads to eosinophil arrest on activated endothelium, extravasation, and continued movement through the bronchial tissue by interaction with the extracellular matrix (ECM). Circulating eosinophils can exist at different activation levels, which include non-activated or pre-activated (sensitized or “primed”). Further, the bloodstream may lack pre-activated cells, due to such eosinophils having arrested on endothelium or extravasated into tissue. Increased expression, and in some instances, decreased expression of cell-surface proteins, including CD44, CD45, CD45R0, CD48, CD137, neuropeptide S receptor, cytokine receptors, Fc receptors, and integrins (receptors mediating cell adhesion and migration by interacting with ligands on other cells or in the ECM), and activated states of integrins or Fc receptors on blood eosinophils have been reported to correlate with aspects of asthma. A subset of these proteins has been reported to respond to intervention, e.g., with anti-interleukin (IL)-5. How these surface proteins and the activation state of the eosinophil respond to other interventions, e.g., with anti-IL-4 receptor alpha or anti-IL-13, is unknown. Eosinophil surface proteins suggested to be biomarkers of activation, particularly integrins, and reports on correlations between eosinophil activation and aspects of asthma are described in this review. Intermediate activation of beta1 and beta2 integrins on circulating eosinophils correlates with decreased pulmonary function, airway inflammation, or airway lumen eosinophils in non-severe asthma. The correlation does not appear in severe asthma, likely due to a higher degree of extravasation of pre-activated eosinophils in more severe disease. Bronchoalveolar lavage (BAL) eosinophils have highly activated integrins and other changes in surface proteins compared to blood eosinophils. The activation state of eosinophils in lung tissue, although likely very important in asthma, is largely unknown. However, some recent articles, mainly on mice but partly on human cells, indicate that tissue eosinophils may have a surface phenotype(s) different from that of sputum or BAL eosinophils.

## Introduction

Asthma is often characterized by eosinophil-rich airway inflammation (1–8). Such eosinophilic inflammation is associated with exacerbations and appears to participate in airway remodeling (1, 8–14). Eosinophil recruitment from the bloodstream depends on circulating blood eosinophils becoming activated, which leads to eosinophil arrest on activated endothelium, extravasation, and continued movement through the bronchial tissue and lumen by interaction with the extracellular matrix (ECM) (2, 8, 15–17). Circulating eosinophils can exist in different states, including non-activated, or pre-activated or “primed” (8, 18). Moreover, the bloodstream may lack pre-activated cells, due to such eosinophils having extravasated (8). Increased expression, and in some instances, decreased expression of cell-surface proteins and activated states of integrins or Fc receptors on blood eosinophils have been reported to correlate with asthma (8, 19). Some of these proteins have been reported to respond to intervention (19).

This review will discuss eosinophil surface proteins proposed to be biomarkers of eosinophil activation and evidence for associations between the activation status of eosinophils and aspects of asthma. The search strategy is described in the notes for Table 1. Further, this review will discuss a subset of these proteins that appears to be downregulated or less activated on circulating eosinophils in severe asthma or after whole-lung antigen challenge. Finally, it will discuss how some eosinophil surface proteins respond to pharmaceutical intervention. A model of eosinophil activation status, focusing on integrins, in the circulation and the airway will also be presented.

**Table 1:** Eosinophil surface proteins altered after antigen challenge or in the airway, or associated with asthma or aspects of asthma.

Protein	Observation	Reference
CD35 (CR1)	Downregulated in bronchoalveolar lavage (BAL)	(20)
CD44	Upregulated after segmental lung antigen challenge, in BAL, or in sputum	(21, 22)
CD45	Upregulated in asthma	(23)
CD45R0	Upregulated in asthma or mild-moderate asthma	(23, 24)
CD48	Upregulated in moderate asthma	(25, 26)
CD58	Upregulated in BAL	(27)
CD63 (lysosome-associated membrane protein 3)	Upregulated in BAL or sputum	(27, 28)
CD66b (CEACAM8)	Upregulated in sputum	(28)
CD66e (CEACAM5)	Upregulated after segmental lung antigen challenge or in BAL	(22)
CD67	Upregulated in BAL	(27)
CD69	Upregulated after whole-lung antigen challenge, in BAL, or in sputum	(28–32)
CD137 (tumor necrosis factor receptor superfamily member 9, induced by lymphocyte activation, 4-1BB)	Upregulated in asthma	(33)
CD274 (programmed death ligand 1)	Upregulated in sputum	(28)
α_L_ integrin (CD11a)	Upregulated in asthma or after segmental lung antigen challenge	(34, 35)
α_M_ integrin (CD11b)	Upregulated after segmental lung antigen challenge, in BAL, or in sputum; Correlates inversely with PC_20_	(20, 27, 28, 34, 36–40)
α_X_ integrin (CD11c)	Upregulated in BAL or sputum	(27, 37)
α_D_ integrin	Upregulated in BAL	(34, 36, 41, 42)
β_2_ integrin (CD18)	Upregulated after segmental lung antigen challenge or in BAL	(34, 36)
Aminopeptidase N (CD13)	Upregulated in BAL	(43)
βc (CD131)	Downregulated in BAL	(44)
FcαRI (CD89)	Upregulated in asthma	(45)
FcγRIII (CD16)	Upregulated in allergic asthma or after whole-lung antigen challenge	(46)
Glucagon-like peptide-1R	Downregulated in allergic asthma	(47)
Granulocyte monocyte-colony stimulating factorRα (CD116)	Upregulated in BAL	(44, 48)
HLA-DR	Upregulated in BAL or sputum	(20, 37)
Intercellular adhesion molecule-1 (CD54)	Upregulated in BAL or sputum	(27, 37)
Interleukin (IL)-2Rα (CD25)	Upregulated in BAL	(22)
IL-3Rα (CD123)	Upregulated after segmental lung antigen challenge or in BAL	(22, 48)
IL-5Rα (CD125)	Downregulated in BAL	(44, 48)
IL-17RA	Upregulated in mild allergic asthma	(49)
IL-17RB	Upregulated in mild allergic asthma	(49)
L-selectin (CD62L)	Downregulated in BAL or sputum	(27, 28, 38, 40, 50)
Neuropeptide S R	Upregulated in severe asthma	(51)
P-selectin glycoprotein ligand-1 (CD162)	Upregulated after segmental lung antigen challenge or (48 h) after whole-lung antigen challenge	(34, 52)
Semaphorin 7A (CD108)	Upregulated in BAL	(53)
Activated α_M_ integrin	Highly activated conformation [reported by monoclonal antibody (mAb) CBRM1/5] in BAL or sputum	(28, 34, 41)
Activated β_1_ integrin (CD29)	Partially activated conformation (reported by mAb N29) increased in all or non-severe asthma, or after segmental antigen challenge	(34, 36, 52, 54, 55)
	Correlates negatively with forced expiratory volume in 1 s (FEV_1_) after or during withdrawal of inhaled corticosteroid (ICS) in non-severe asthma and predicts decreased FEV_1_ according to receiver–operator characteristic analysis	
	Correlates with fraction of exhaled nitric oxide (FENO) upon withdrawal of ICS in non-severe asthma	
	Correlates negatively with FEV_1_/forced vital capacity in younger non-severe asthmatic patients or in phenotype clusters 1–2 (mild–moderate allergic asthma)	
	At 48 h, post-segmental lung antigen challenge correlates with decrease in FEV_1_ during the late phase post-whole-lung antigen challenge in mild allergic asthma	
	Highly activated conformation (reported by mAbs HUTS-21 and 9EG7) in BAL	
Activated β_2_ integrin	Partially activated conformation (reported by mAb KIM-127) correlates with BAL eosinophil percentage in mild allergic asthma	(34, 36)
	Highly activated conformation (reported by mAb24) in BAL	
Activated FcγRII	Activated conformation (reported by mAb A17 or A27) increased in mild asthma, after whole-lung antigen challenge (in dual responders), or in BAL	(18, 56, 57)
	Correlates with FENO in asthma	

*Observations refer to expression level, usually determined by flow cytometry, and are, if not indicated otherwise, on human blood eosinophils. For abbreviations, please see list immediately after abstract*.

*The search strategy used Pubmed (https://www.ncbi.nlm.nih.gov/pubmed?db=PubMed) with various combinations of terms including “eosinophils” and “activation” and “asthma” and “state” or “status” or “biomarker” or “review” (the combination of only “eosinophils” and “activation” and “asthma” resulted in an unmanageable large number of publications). Publications covering years until 2017 were examined, with primary publications covering the years 2014–2017 being especially examined. Primary references covering years until 2013 were partly obtained from published review articles*.

## Eosinophil Surface Proteins Altered after Antigen Challenge, in the Airway, or in Asthma

Upregulation or downregulation of eosinophil surface proteins and activated conformations of integrins and Fc receptors have been proposed to be biomarkers of eosinophil activation, in many cases due to the reaction of eosinophils to various stimuli *in vitro* (2, 8, 17–19, 58–61). Many, but not all, i.e., not all that change in response to *in vitro* stimulation, of these surface proteins have been reported to be altered on blood eosinophils after whole-lung or segmental lung antigen challenge, or on bronchoalveolar lavage (BAL), or sputum eosinophils (Table 1). In addition, the surface proteins may be altered on blood eosinophils in asthma or in a manner that correlates with features of asthma (Table 1) (19). Segmental and whole-lung antigen challenge are models of allergic airway inflammation (62) and asthma exacerbation (63), respectively. Up- or downregulation in Table 1 refers to changed or different protein expression of a cell surface protein, which usually has been determined by flow cytometry. Further, alterations are listed independently of what the mechanism may be, e.g., translocation to the surface from intracellular granules or the effect of increased transcription or protein synthesis and may consist of an alteration in mean or median expression on all eosinophils or an alteration in the percentage of expressing eosinophils (8, 19). Some references have studied purified cells, while others have used whole blood, BAL, or sputum cells. An unfractionated sample is beneficial in that just a small volume or number of cells is needed and that isolated cells *in vitro* may be different and more activated than cells *in vivo* (8, 19, 64, 65). Regarding more detailed information about individual proteins, please see Ref. (8).

Several proteins, including CD45, CD45R0, CD48, CD137, IL-17 receptor (R) A and B, α_L_ integrin, and some of the Fc receptors, are increased or decreased on circulating eosinophils in asthma compared to normal, non-allergic healthy individuals (Table 1) (8, 19). One specific example is that IL-17R and B (subunits of IL-25R) are increased in patients with non-severe allergic asthma but not in non-asthmatic patients with atopy (8, 19, 49). In the case of some proteins, reports are conflicting. For instance, some workers reported FcγRIII (CD16) to be increased on blood eosinophils in allergic asthma (or allergic rhinitis) (46) (Table 1), while other authors reported no alterations in airway allergies when compared to control subjects (8, 19, 66).

The expression level of a particular protein may not only be an effect of the eosinophil having been exposed to cytokines or other stimuli but may also partly result from actions of regulatory factors *in vivo*. One recent example of such a factor that may regulate eosinophil activation is glucagon-like peptide (GLP)-1, a member of the incretin family of hormones, which regulates glucose metabolism (47). A GLP-1 analog inhibited upregulation of α_M_ integrin and CD69 *in vitro* in response to lipopolysaccharide (47). Further, expression of GLP-1 receptor was lower on blood eosinophils in patients with allergic asthma than in normal controls (Table 1). The lower level of GLP-1R in asthma than in healthy subjects indicates that the eosinophil response to activating stimuli may be more regulated by GLP-1 in healthy persons and that eosinophil activation may be more easily achieved in asthma than in health. Further, Mitchell and colleagues suggest that GLP-1 agonists may have additional indications in treating patients with concomitant type 2 diabetes mellitus and asthma (47).

## Associations with Aspects of Asthma

Expression and activation of some proteins have been found to be associated with clinical findings of asthma (Table 1) (8, 17, 19). Activated β_1_ integrin, specifically the intermediate-activity conformation recognized by monoclonal antibody (mAb) N29, on blood eosinophils correlates inversely with lung function in non-severe asthma (52, 54), or directly with the magnitude of the late-phase reaction in mild allergic asthma (36) or with exhaled NO [fraction of exhaled nitric oxide (FENO)], which reports airway inflammation, after inhaled corticosteroid (ICS) withdrawal (54). In addition, by receiver–operator characteristic (ROC) analysis, β_1_ integrin activation, assessed with N29, predicts lowered pulmonary function in mild asthmatic patients (54). Intermediate-activity β_2_, reported by the antibody KIM-127, is associated with the percentage of BAL eosinophils in patients with mild allergic asthma (34). Finally, activation of FcγRII (CD32) correlates with FENO in asthma (18).

## Downregulation in Severe Asthma or after Antigen Challenge

Some surface proteins on blood eosinophils are downregulated in more severe or uncontrolled asthma compared to less severe disease (Table 2). These include CD44, a hyaluronan receptor, and CD48, whose levels are lower in poorly controlled or severe asthma compared to well-controlled or moderate disease (21, 25). Similarly, activated β_1_ integrin, reported by mAb N29 (see above), is increased in non-severe, but not in severe, asthma compared to healthy control subjects (52).

**Table 2:** Eosinophil surface proteins downregulated in severe or poorly controlled asthma, or transiently after whole-lung antigen challenge.

Protein	Observation	Reference
CD44	Downregulated in poorly controlled compared to well-controlled asthma	(21)
CD48	Downregulated in severe compared to moderate asthma	(25)
P-selectin (CD62P)	Decreased transiently after whole-lung antigen challenge	(52)
P-selectin glycoprotein ligand-1 (CD162)	Decreased transiently after whole-lung antigen challenge	(52)
Activated β_1_ integrin	Intermediate-activity state (recognized by monoclonal antibody N29) increased in non-severe but not severe asthma	(52)
	Decreased transiently after whole-lung antigen challenge	

*Observations refer to expression level, determined by flow cytometry, and are on human blood eosinophils*.

*P-selectin is not synthesized by eosinophils (67) but can be associated with the eosinophil surface and is likely derived from activated platelets (52, 64)*.

A possible explanation for this phenomenon is a high degree of ongoing extravasation of the most activated eosinophils, i.e., those with the highest level of CD44, CD48, and β_1_ integrin activation, in severe asthma. This is consistent with a role for CD44 in the movement of eosinophils to the airway in mice after antigen challenge (8, 68). Also, CD44, like P-selectin glycoprotein ligand (PSGL)-1, relocalizes on blood eosinophils after stimulation with IL-5 or related cytokines, when the eosinophil changes shape and polarizes, and becomes concentrated at one end of the eosinophil in the nucleopod, which is a specialized uropod next to the nucleus (69). Such clustering of CD44 and other surface molecules may stimulate arrest and extravasation of eosinophils (8, 69). Similarly, uropod elongation, at the rear of a moving cell, is considered a crucial step in other leukocytes, including neutrophils and lymphocytes, before extravasation (70). Further, there is greater lung endothelial expression of vascular cell adhesion molecule (VCAM)-1, the ligand for α_4_β_1_ integrin, in severe asthma, as observed in bronchial biopsies (71), which is compatible with efficient extravasation of eosinophils with activated α_4_β_1_ integrin. The fraction of eosinophils that does not adhere to VCAM-1 *in vitro* has decreased β_1_ activation, as reported by N29 (64), which also provides support for the scenario in which the eosinophils with a higher degree of α_4_β_1_ activation are the ones that preferentially adhere to VCAM-1 (8, 17, 64). Finally, N29 reactivity, surface-associated P-selectin, and level of PSGL-1 decrease transiently after whole-lung antigen challenge in patients with mild allergic asthma (Table 2) (52). P-selectin activates eosinophil β_1_ integrin and induces the N29 epitope *in vitro* (64) and is associated with N29 reactivity *in vivo* (52). P-selectin is not synthesized by eosinophils (67). The P-selectin bound to the eosinophil surface is likely derived from activated platelets associated with the eosinophils; a proportion (variable among different subjects) of eosinophils both in whole blood samples and purified eosinophils stain positively for the platelet marker α_IIb_ integrin (CD41) and P-selectin by flow cytometry or immunofluorescence microscopy (52, 64). The role of platelets, platelet activation, and platelet–eosinophil complexes in eosinophil recruitment and eosinophilic inflammation is the focus of another review within this Frontiers in Medicine Research Topic of “Pathogenic Advances and Therapeutic Perspectives for Eosinophilic Inflammation” and is described in more detail there (Shah S, Page CP, and Pitchford S: “Platelet–eosinophil interactions as a potential therapeutic target in allergic inflammation and asthma,” submitted). Overall, the observations described above support the scenario that the most activated eosinophils; i.e., in this case, the cells with the highest degree of bound P-selectin, the highest level of the P-selectin counter-receptor PSGL-1, and activated α_4_β_1_; extravasate, for instance, after whole-lung antigen challenge or in severe asthma.

In addition to possible extravasation of the most activated eosinophils in severe asthma and after whole-lung antigen challenge, it may also be, at least under some circumstances, that it is the most “activatable” cells that extravasate and the least “activatable” ones that remain in the circulation. High percentage of sputum eosinophils in asthmatic patients was found to be associated with low or no upregulation of α_M_ integrin or activation of FcγRII (CD32) on blood eosinophils in response to formylmethionine-leucyl-phenylalanine (fMLF) *in vitro*, whereas low sputum eosinophil count was associated with great α_M_ upregulation and CD32 activation in response to fMLF (72). These results indicate that the responsiveness of circulating eosinophils to a chemoattractant is lower in subjects with high sputum eosinophilia. This is possibly because the most responsive cells are continuously extravasating. An alternative or additional explanation may be that in patients with low sputum eosinophilia, the circulating eosinophils are not activated and are able to mount a great response to fMLF. On the other hand, in subjects with high sputum eosinophilia, the blood cells may already be at least partly activated (i.e., α_M_ already upregulated and CD32 altered to an activated conformation). In such a situation, fMLF may not achieve, or may achieve only little, further activation *in vitro*.

## Response to Intervention

**Figure 1:**
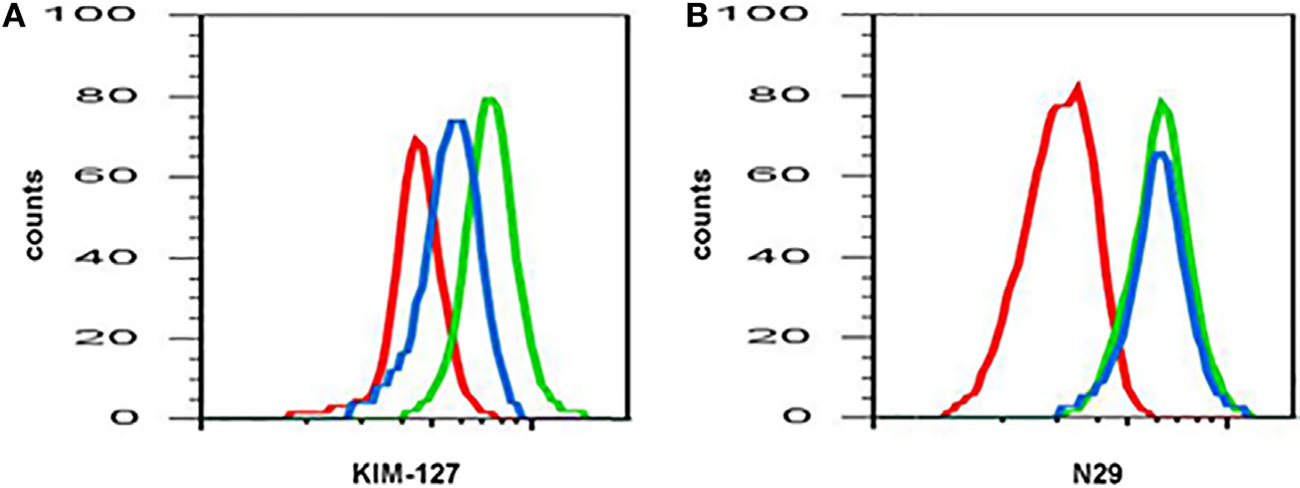
Anti-interleukin (IL)-5 (mepolizumab) decreases β_2_ but not β_1_ integrin activation on blood eosinophils. Reactivity of monoclonal antibody **(A)** KIM-127 (to the intermediate-activity state of β_2_ integrin), and **(B)** N29 (to the intermediate-activity state of β_1_ integrin) on blood eosinophils before (green) and after (blue) anti-IL-5 mepolizumab administration. Red, isotype control. A representative subject with mild allergic asthma from Ref. (34).

The expression or activation state of some proteins changes after pharmaceutical administration, e.g., with mepolizumab, an antibody against IL-5 (Table 3) (19). Anti-IL-5 mepolizumab causes decreased β_2_ integrin, but not β_1_ integrin, activation of blood eosinophils (Figure 1) (34). This indicates that the intermediate β_2_ activation state on circulating eosinophils is the result of exposure to IL-5 *in vivo* and is consistent with *in vitro* data that IL-5 causes β_2_ but not β_1_ activation, whereas P-selectin activates β_1_ but not β_2_ (64). The differential response to anti-IL-5 demonstrates that pharmaceutical intervention may inhibit one aspect of eosinophil activation but not another. Further, comparing blood eosinophils after segmental lung antigen challenge pre- and post-mepolizumab demonstrated that anti-IL-5 caused decreased levels of α_L_, α_M_, and β_2_ integrins as well as PSGL-1 (34), indicating that the upregulation of these proteins that occurs on blood eosinophils after segmental lung antigen challenge is IL-5-dependent. Finally, in contrast to the situation with blood eosinophils, anti-IL-5 did not affect the activation state of α_M_β_2_ and the level of α_L_, α_M_, and β_2_ on BAL eosinophils (34), indicating that the activation status of airway lumen eosinophils is independent of IL-5. This is consistent with the finding that BAL eosinophils have downregulated or no IL-5 receptor (Table 1) (44, 48), whereas they, in contrast, have upregulated IL-3 and granulocyte monocyte-colony stimulating factor (GM-CSF) receptors (Table 1) (22, 44, 48).

**Table 3:** Eosinophil surface proteins reported to respond to intervention in asthma.

Protein	Observation
α_L_ integrin (CD11a)	Decreased by anti-interleukin (IL)-5 (mepolizumab) after segmental lung antigen challenge
α_M_ integrin (CD11b)	Decreased by anti-IL-5 (mepolizumab) after segmental lung antigen challenge
β_2_ integrin (CD18)	Decreased by anti-IL-5 (mepolizumab) after segmental lung antigen challenge
P-selectin glycoprotein ligand-1 (CD162)	Decreased by anti-IL-5 (mepolizumab) after segmental lung antigen challenge
Activated β_2_ integrin	Intermediate-activity state (recognized by monoclonal antibody KIM-127) decreased by anti-IL-5 (mepolizumab)

*Observations refer to expression level, determined by flow cytometry, and are on blood eosinophils in mild allergic asthma (34)*.

Whereas anti-IL-5 causes a decrease in blood eosinophil count (9, 10, 34), administration of anti-IL-13 or anti-IL-4 receptor α causes an increase in blood eosinophils (11, 73, 74). This observation is consistent with a scenario in which IL-13- or IL-4-induced factors, including VCAM-1, periostin, and eotaxins, promote eosinophil extravasation and trafficking (75–78). It would be interesting to know whether the circulating eosinophils after anti-IL-13/IL-4Rα treatment have become more or less activated, or not altered, but the surface phenotype of blood eosinophils after anti-IL13/IL-4Rα has not yet been reported. On one hand, one may imagine that they should become more activated, since ongoing extravasation of activated cells presumably has decreased, so activated cells instead may be expected to accumulate in the circulation. On the other hand, IL-13 or IL-4 themselves may stimulate some aspects of eosinophil activation, e.g., they have been reported to upregulate CD69 (8), indicating that anti-IL-13/IL-4Rα may lead to lower activation of blood eosinophils, or that at least some aspects of eosinophil activation may be decreased.

Recently, a mathematical modeling approach was taken in order to understand the effect of anti-IL therapy on eosinophil activation and dynamics (79). The mathematical model of Karelina and colleagues predicts a rapid decrease in total and activated eosinophil counts in blood and airways after anti-IL-5 mepolizumab administration. The decrease in blood eosinophils in the model is consistent with the literature, whereas the model appears to predict a greater proportional decrease in airway eosinophil counts than what actually happens after mepolizumab (9, 10, 34). The decrease in blood eosinophil activation in the model is consistent with the observed decrease in β_2_ integrin activation (see here above and Figure 1) (34). However, the decrease in activated eosinophils in the airway in the model is not consistent with the observed lack of effect on integrin activation and expression levels on BAL eosinophils after mepolizumab (34). This disagreement between the model and observations may be because the model assumes that IL-5 is involved in the activation of airway eosinophils, whereas in reality, it may not be. Other factors such as IL-3 may be essential or compensate for IL-5 to stimulate and maintain airway eosinophil activation (see above and more below). Further, the model correctly predicts an increase in total eosinophils in blood and a decrease in the airway for anti-IL-13 therapies. Finally, it also predicts an increase in the number of activated eosinophils in blood after anti-IL-13, something which has not been reported but would be interesting to investigate, as mentioned above.

## A Model for Eosinophil Activation States in the Circulation and the Airway in Asthma

**Figure 2:**
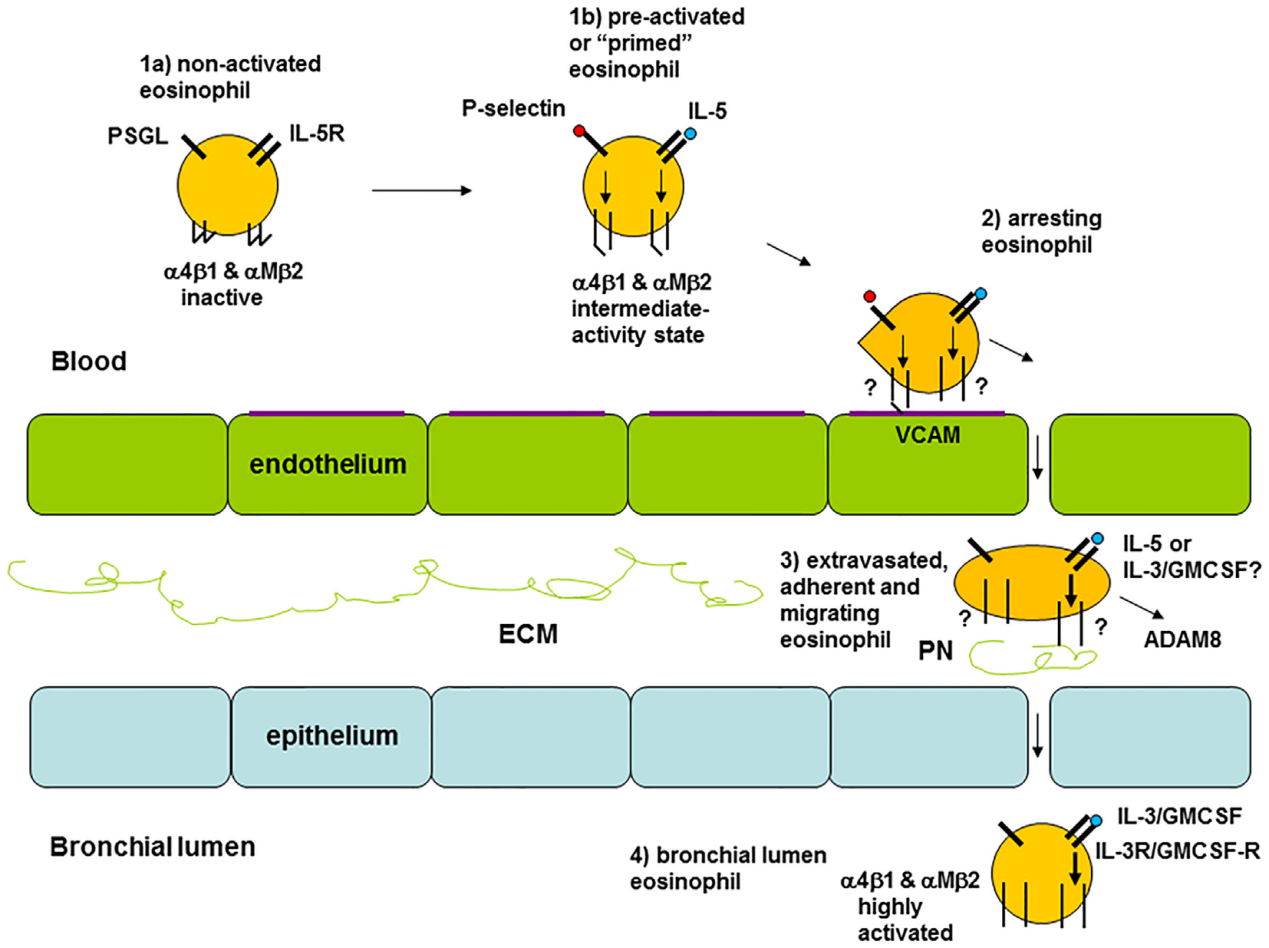
Model of eosinophil activation states in asthma. (1a) Circulating non-activated eosinophil with α_4_β_1_ and α_M_β_2_ integrins in the inactive conformation or state, as found in normal subjects, some patients with non-severe asthma, or as observed in severe asthmatic patients likely because of a high degree of extravasation of activated eosinophils. (1b) Pre-activated or “primed,” partly activated, circulating eosinophil with α_4_β_1_ and α_M_β_2_ in the intermediate-activity state, as a result of signaling triggered by P-selectin (likely derived from activated platelets, see the main text) and low concentration of interleukin (IL)-5, respectively, as found primarily in some subjects with non-severe asthma. (2) Eosinophil arresting on activated endothelium in asthma with α_4_β_1_ and α_M_β_2_ in unknown state, with α_4_β_1_ primarily mediating arrest on vascular cell adhesion molecule (VCAM)-1 with a possible minor contribution of α_M_β_2_. (3) Extravasated, adherent, and migrating tissue eosinophil in asthma with α_4_β_1_ and α_M_β_2_ likely in the high-activity state, with high-activity α_M_β_2_, resulting from cytokine-triggered signaling, mediating interaction with the adhesive and pro-migratory extracellular matrix (ECM) protein periostin, and the eosinophil-releasing disintegrin and metalloproteinase (ADAM) 8 involved in PN degradation and cell migration. (4) Bronchial lumen highly activated eosinophil in asthma with α_4_β_1_ and α_M_β_2_ in the high-activity state and with downregulated or no IL-5 receptor, and with high-activity α_M_β_2_ resulting from IL-3 and/or granulocyte monocyte-colony stimulating factor (GM-CSF)-triggered signaling. Modified and extended from Ref. (8) and also based on Ref. (34, 36, 44, 69, 80). Note: this model focuses on the activation states of integrins and on receptors for IL-5 family cytokines. It is not intended to be a full rendition of all possible factors involved in eosinophil recruitment. For instance, glycoproteins and glycans, including endothelial surface selectins and their role in eosinophil rolling, are covered in other reviews within this Research Topic (O’Sullivan JA, Carroll DJ, and Bochner BS: “Glycobiology of eosinophilic inflammation: contributions of siglecs, glycans, and other glycan-binding proteins,” submitted; and Rao AP, Ge XN, and Sriramarao P: “Regulation of eosinophil recruitment and activation by galectins in allergic asthma,” accepted). Further, chemokines and their receptors are the focus of yet another review (Larose M-C, Archambault A-S, Provost V, Laviolette M, and Flamand N: “Regulation of eosinophil and group 2 innate lymphoid cell trafficking in asthma,” submitted).

The results from the studies on integrins discussed above suggest that there is variation in the activation status of circulating eosinophils among different individuals (8). Healthy persons and some patients with asthma display inactive β_1_ integrins, patients with non-severe asthma have partially activated β_1_ and β_2_ integrins to different degrees, and patients with severe asthma have inactive or less activated β_1_ integrins (8). The latter occurs likely due to arrest and extravasation of activated eosinophils (see model in Figure 2 and references in the figure legend). Similarly, a scenario for FcγRII (CD32) activation on blood eosinophils has been described, where the degree of activation initially is elevated along with a higher level of systemic inflammation and then lower at the greatest degree of systemic inflammation (8, 18).

Airway lumen eosinophils, as sampled during BAL, have highly activated and upregulated α_M_β_2_ and highly activated β_1_ (34, 36), downregulated or no IL-5 receptor (44, 48), as well as upregulated IL-3 receptor (22, 48) and upregulated and highly activated FcγRII (CD32) (56). As the integrin activation state and levels on BAL eosinophils are not affected by anti-IL-5 (see above in Section “Response to Intervention”) and BAL eosinophils lack IL-5-receptor, the airway lumen eosinophil phenotype is presumably the result of and maintained by other stimuli than IL-5, e.g., the related cytokines IL-3 and/or GM-CSF. IL-3 is the most likely responsible factor, since it, compared to IL-5, causes a higher degree of prolonged upregulation and activation of α_M_β_2_ and CD32 (81).

The activation status of the lung tissue eosinophil in asthma is largely unknown. As depicted in Figure 2, eosinophils in lung tissue likely are adherent to or migrating in the ECM, e.g., by interacting with the ECM protein periostin, which is upregulated and associated with eosinophil recruitment to the airway in type 2 immunity-high asthma (82–86). Eosinophil adhesion to and motility on periostin is mediated by α_M_β_2_ integrin and stimulated by nanogram per milliliter IL-5 (75, 80), which induces the high-activity conformation of α_M_β_2_ (41, 64). Thus, assuming that tissue eosinophils interact with periostin, they likely have highly activated α_M_β_2_. Whether tissue eosinophils express (like blood eosinophils) or lack (like BAL eosinophils) IL-5R, and in the latter case are stimulated and maintained active by GM-CSF or IL-3, appears uncertain and would be very interesting to determine.

Some recent very interesting articles studied mouse lung tissue eosinophils and partly also human lung tissue eosinophils (87, 88). Abdala Valencia and colleagues reported that, after antigen challenge, mouse lung tissue eosinophils shifted from a surface phenotype with intermediate expression of Siglec-F and no or very low α_X_ integrin (CD11c) to a Siglec-F-high/CD11c-low phenotype, and that BAL eosinophils were of the latter phenotype (87). Mesnil and others found that mouse steady-state pulmonary resident eosinophils were IL-5-independent and expressed an intermediate level of Siglec-F (in consistency with the first tissue phenotype in the Abdala Valencia publication), high L-selectin (CD62L), and low CD101 (immunoglobulin superfamily member 2) (88). After antigen challenge, these resident tissue cells were accompanied by newly recruited inflammatory tissue eosinophils, which were IL-5-dependent, Siglec-F-high, CD62L-low, and CD101-high (88, 89). In addition, Mesnil and colleagues determined that parenchymal resident eosinophils found in non-asthmatic human lungs had a CD62L-high, IL-3R-low phenotype, which was distinct from the phenotype of asthmatic sputum eosinophils, being CD62L-low/IL-3R-high (88). Their description of sputum eosinophils is consistent with earlier findings on sputum and BAL eosinophils (Table 1). Thus, both in humans and mice, lung tissue eosinophils may be of two phenotypes, one resident phenotype unrelated to asthma and different from the asthmatic airway lumen eosinophil phenotype, and one inflammatory phenotype recruited in asthma and similar or more similar to the airway lumen phenotype. A more detailed description of the tissue-resident eosinophils is given in another review within this Research Topic (Marichal T, Mesnil C, and Bureau F: “Homeostatic eosinophils: characteristics and functions,” submitted). As indicated above, a more complete description of the inflammatory lung tissue eosinophil phenotype in asthma and a comparison to the blood and airway lumen phenotypes in asthma are warranted, e.g., to answer questions about integrin activation status of the lung tissue eosinophils and whether they express IL-5R.

## Conclusion and Perspectives

In this article, a number of proteins on the cell surface that have been suggested to mark eosinophil activation and are altered after antigen challenge or in the airway, or are associated with asthma or aspects of asthma, as well as a subset of these proteins that respond to intervention are reviewed. Partial β_1_ integrin activation on blood eosinophils is associated with impaired pulmonary function or airway inflammation, and partial β_2_ integrin activation is associated with airway eosinophilia in non-severe asthma. The associations do not occur in severe asthma, presumably due to greater extravasation of pre-activated eosinophils in severe disease. Airway lumen eosinophils have highly activated integrins and other changes in surface proteins compared to blood eosinophils. The activation state(s) of eosinophils in human lung tissue, although likely very important in asthma, is largely unknown but has begun to be studied.

The utility of the potential biomarkers of eosinophil activation in blood, a clinically accessible compartment, e.g., as correlators with or reporters of aspects of asthma, particularly severe asthma, needs to be explored further in translational and clinical studies. Although an occasional marker increases with asthma severity, some markers are downregulated in severe disease compared to non-severe disease. Possible reasons for the latter phenomenon are discussed above in the text. Still, since some of the markers respond to anti-IL-5, these or other IL-5-dependent markers may be predictors of response to intervention. The effect of other therapies, e.g., anti-IL-13/IL-4R, on surface markers of eosinophil activation has not been reported but is a very interesting question. For instance, it may be interesting to examine whether potential alterations in eosinophil surface activation markers after various interventions may turn out to be associated with disease improvement, or possibly with decreased or increased risks for adverse eosinophil-related events. Finally, the potential relevance of these biomarkers in other eosinophilic and allergic diseases (19) also requires future exploration.

## Author Contributions

MJ conceived and designed, and interpreted the literature for this review; drafted and revised the manuscript, and approved the final version.

## Conflict of Interest Statement

MJ received a fee for consulting from Guidepoint Global, a fee from Genentech for speaking, and funds for research from Hoffmann-La Roche; and is an advisory board member for Genentech.

## Abbreviations

ADAM, a disintegrin and metalloproteinase; BAL, bronchoalveolar lavage; βc, common β chain (of IL-3, IL-5, and GM-CSF receptors); C, complement; CD, cluster of differentiation; ECM, extracellular matrix; Fc, fragment, crystallizable (of immunoglobulin); FENO, fraction of exhaled nitric oxide; FEV_1_, forced expiratory volume in 1 s; FVC, forced vital capacity; GLP, glucagon-like peptide; GM-CSF, granulocyte macrophage-colony stimulating factor; HLA, human leukocyte antigen; ICAM, intercellular adhesion molecule; IGSF, immunoglobulin superfamily member; IL, interleukin; ILA, induced by lymphocyte activation; LAMP, lysosome-associated membrane protein; LPS, lipopolysaccharide; mAb, monoclonal antibody; PC_20_, provocative concentration of methacholine or histamine producing a 20% fall in FEV_1_; PD-L, programmed death ligand; PSGL, P-selectin glycoprotein ligand; R, receptor; ROC, receiver–operator characteristic; TNFRSF, tumor necrosis factor receptor superfamily member; VCAM, vascular cell adhesion molecule.
